# Crystal structure of *catena*-poly[[[bis­(3-oxo-1,3-di­phenyl­prop-1-enolato-κ^2^ *O*,*O*′)zinc(II)]-μ_2_-tris­[4-(pyridin-3-yl)phen­yl]amine-κ^2^ *N*:*N*′] tetra­hydro­furan monosolvate]

**DOI:** 10.1107/S2056989019012350

**Published:** 2019-09-10

**Authors:** Yukiyasu Kashiwagi, Koji Kubono, Toshiyuki Tamai

**Affiliations:** a Osaka Research Institute of Industrial Science and Technology, 1-6-50 Morinomiya, Joto-ku, Osaka 536-8553, Japan; b Osaka Kyoiku University, Kashiwara, 536-8553, Japan

**Keywords:** crystal structure, coordination polymer, tri­aryl­amine, *β*-diketonato zinc(II), C—H⋯π inter­actions

## Abstract

The reaction of bis­(3-oxo-1,3-di­phenyl­prop-1-enolato-κ^2^ *O*,*O*′)zinc(II), [Zn(dbm)_2_], with tris­[4-(pyridin-3-yl)phen­yl]amine (T3PyA) in tetra­hydro­furan (THF) afforded the title crystalline coordination polymer, {[Zn(C_15_H_11_O_2_)_2_(C_33_H_24_N_4_)]·C_4_H_8_O}_*n*_. The asymmetric unit contains two independent halves of Zn(dbm)_2_, one T3PyA and one THF. Each Zn^II^ atom is located on an inversion centre and adopts an elongated octa­hedral coordination geometry, ligated by four O atoms of two dbm ligands in equatorial positions and by two N atoms of pyridine moieties from two different bridging T3PyA ligands in axial positions. The crystal packing shows a one-dimensional polymer chain in which the two pyridyl groups of the T3PyA ligand bridge two independent Zn atoms of Zn(dbm)_2_. In the crystal, the coordination polymer chains are linked *via* C—H⋯π inter­actions into a sheet structure parallel to (010). The sheets are cross-linked *via* further C—H⋯π inter­actions into a three-dimensional network. The solvate THF mol­ecule shows disorder over two sets of atomic sites having occupancies of 0.631 (7) and 0.369 (7).

## Abstract 2

The synthesis and crystal structure of a coordination polymer composed of *β*-diketonato zinc(II) [Zn(dbm)_2_] and tri­aryl­amine-based bridging ligands (T3PyA) is reported. The asymmetric unit in the crystal consists of two independent halves of Zn(dbm)_2_, one T3PyA and one solvate THF. Each Zn^II^ atom is located on an inversion centre and adopts an elongated octa­hedral coordination geometry. In the crystal, the coordination polymer chains are linked *via* C—H⋯π inter­actions into a three-dimensional framework.

## Chemical context   

The structure of coordination polymers generated from the self-assembly of metal ions and bridging organic ligands depends on the mol­ecular structures of the ligands and the coordination geometries of the metal ions. The pyridyl-group-terminated spacer ligands with coordinating ability and optical or electronic functionalities have been widely used to construct a variety of coordination polymers with designable structures and attractive potential applications in material science (Robin & Fromm, 2006 ▸; Allendorf *et al.*, 2009 ▸; Stavila *et al.*, 2014 ▸). Tri­phenyl­amine-based structures are some of the most important moieties and electron-donating groups in organic electronic materials, *e.g*. organic or organic–inorganic hybrid light-emitting diodes and solar cells, because of their electroactivity, photoactivity and chemical stability (Shirota & Kageyama, 2007 ▸; Mahmood, 2016 ▸; Agarwala & Kabra, 2017 ▸). One of the pyridyl-group-terminated tri­phenyl­amine derivatives, tris­[4-(pyridin-3-yl)phen­yl]amine (T3PyA), was firstly synthesized by Hu *et al.* (2013 ▸) as a pH-sensitive fluoro­phore. Recently, its Pd^II^ complex was also reported (Wang *et al.*, 2016 ▸). We report herein on the crystal structure of the title coordination polymer composed of an *exo*-tridentate tripyridyl-type ligand, a *β*-diketonato ligand and a closed-shell Zn^II^ atom as the building blocks.
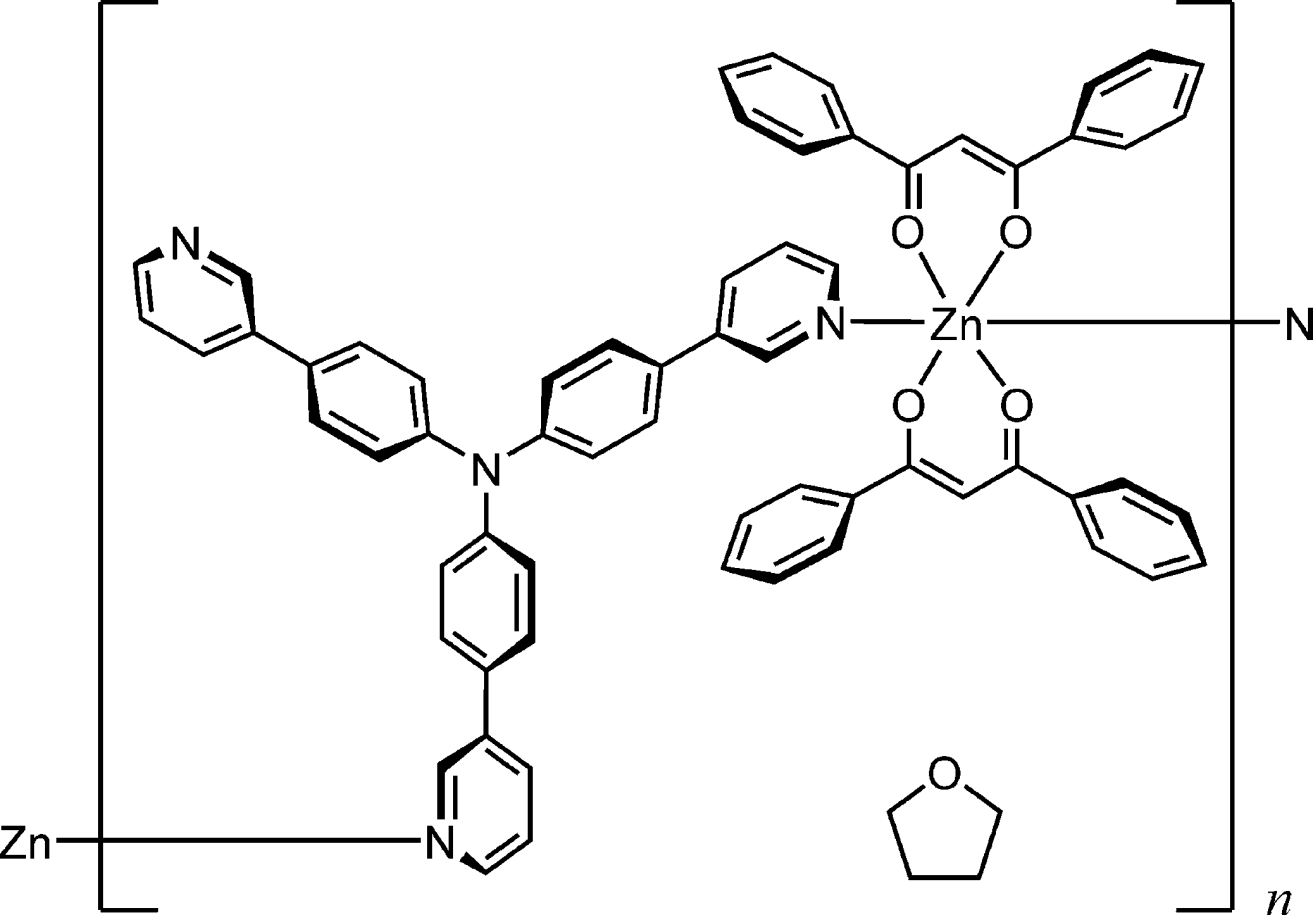


## Structural commentary   

**Figure 1:**
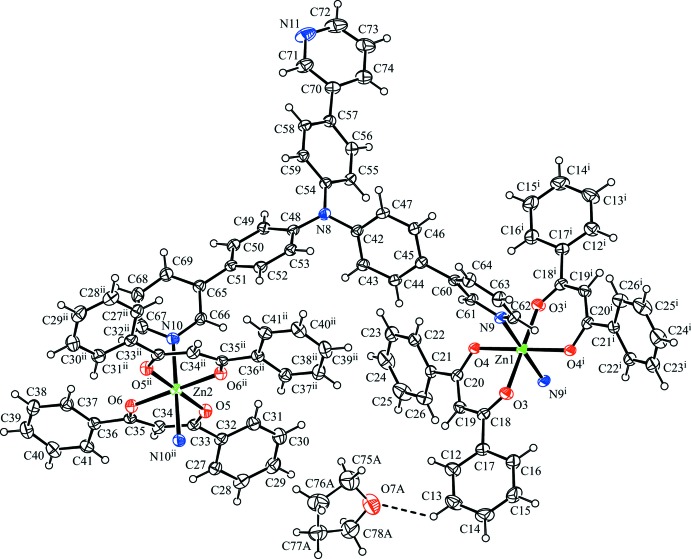
The mol­ecular structure of the title compound, with the atom labelling. Displacement ellipsoids are drawn at the 50% probability level. H atoms are represented by spheres of arbitrary radius. The inter­molecular C—H⋯O hydrogen bond is shown as a dashed line. The minor component of the disordered THF mol­ecule has been omitted for clarity. [Symmetry codes: (i) −*x* + 1, −*y* + 1, −*z* + 2; (ii) −*x*, −*y* + 1, −*z* + 1.]

The asymmetric unit of the title coordination polymer is composed of two unique halves of the bis­(3-oxo-1,3-di­phen­yl­prop-1-enolato-κ^2^ *O*,*O′*)zinc(II) [Zn(dbm)_2_] moiety, one T3PyA ligand bridging the Zn atoms in a μ_2_-κ^2^ mode and one tetra­hydro­furan (THF) solvate mol­ecule (Fig. 1 ▸). Each Zn atom is located on an inversion centre and adopts an elongated octa­hedral coordination geometry, ligated by four O atoms of bidentate *β*-diketonato dbm ligands in equatorial positions and by two N atoms of pyridine moieties from two different bridging T3PyA ligands in axial positions. The equatorial Zn—O bond lengths [2.0440 (17)–2.0629 (18) Å] are shorter than the axial Zn—N bond lengths [Zn1—N9 = 2.199 (2) Å and Zn2—N10 = 2.238 (2) Å]. In the two independent Zn(dbm)_2_ moieties, the bond lengths and angles are similar, but a difference in the dihedral angles between the mean planes of the benzene rings in dbm is observed [56.19 (16)° between the C12–C17 and C21–C26 rings in the moiety containing Zn1, and 30.68 (14)° between the C27–C32 and C36–C41 rings in the moiety containing Zn2]. The bridging T3PyA ligand has three pyridyl N atoms (N9, N10 and N11). Atoms N9 and N10 each coordinate to two different Zn atoms, while atom N11 does not inter­act with the surrounding atoms. The central N atom (N8) of T3PyA shows no pyramidalization, with a displacement of 0.052 (2) Å from the plane of the bonded C atoms (C42, C48 and C54) in the benzene rings. The dihedral angles between the mean planes of the benzene and pyridine rings in T3PyA are 47.56 (13), 33.60 (13) and 26.35 (15)°, respectively, between the C42–C47 and N9/C60–C64 rings, the C48–C53 and N10/C65–C69 rings, and the C54–C59 and N11/C70–C74 rings.

## Supra­molecular features   

**Figure 2:**
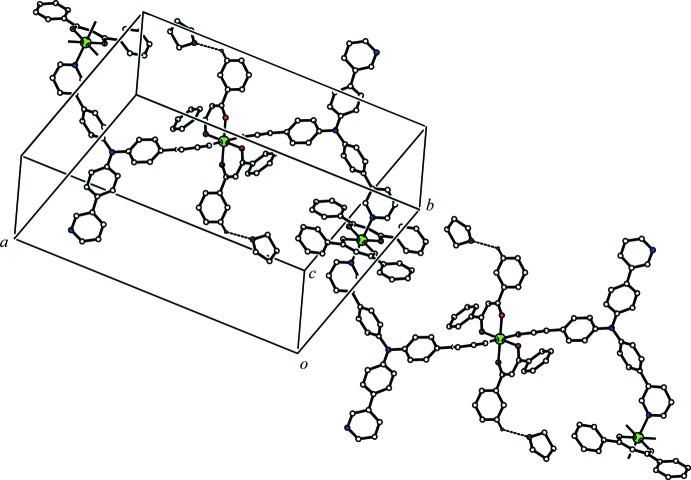
A packing diagram of the title compound, showing a zigzag one-dimensional coordination polymer and solvate THF mol­ecules with the major disordered component. The C—H⋯O hydrogen bonds are shown as dashed lines. H atoms not involved in the inter­actions have been omitted for clarity.

**Figure 3:**
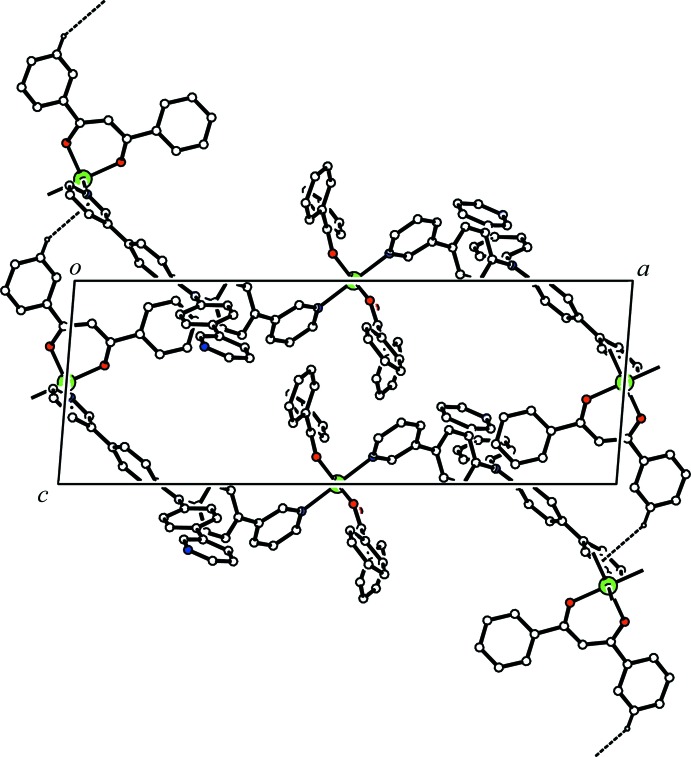
A packing diagram of the title compound viewed along the *b* axis, showing the network sheet structure. The C—H⋯π inter­actions between the coordination polymer chains related by translation along the *c* axis are shown as dashed lines. H atoms not involved in the inter­actions and all components of the disordered THF mol­ecule have been omitted for clarity.

**Figure 4:**
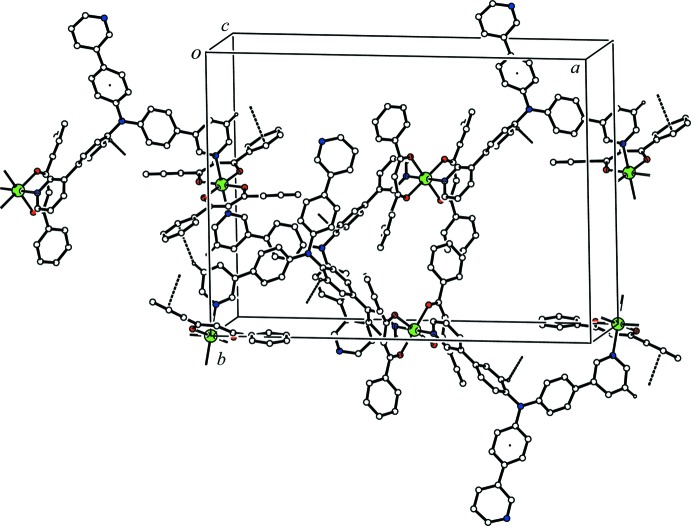
A packing diagram of the title compound, showing the network structure between the coordination polymer chains related by a *c-*glide plane. The C—H⋯π inter­actions are shown as dashed lines. H atoms not involved in the inter­actions and all components of the disordered THF mol­ecule have been omitted for clarity.

In the crystal, the two independent Zn(dbm)_2_ moieties and the bridging T3PyA ligand form a zigzag one-dimensional coordination polymer along [101] (Fig. 2 ▸). There is a C—H⋯O hydrogen bond between the coordination polymer and the major disorder component of the solvate THF mol­ecule (C13—H13⋯O7*A*, Table 1 ▸), while a C—H⋯π inter­action is observed between the minor disorder component of the solvate THF mol­ecule and the coordination polymer (C75*B*—H75*C*⋯*Cg*4^ii^; *Cg*4 is the centroid of the N11/C70–C74 ring; symmetry code as in Table 1 ▸). The coordination polymer chains related by translation along the *c* axis are linked *via* a C—H⋯π inter­action (C40—H40⋯*Cg*1^i^; *Cg*1 is the centroid of the N10/C65–C69 ring; symmetry code as in Table 1 ▸) into a network sheet parallel to (010) (Fig. 3 ▸). In addition, the coordination polymer chains related by a *c-*glide plane are linked *via* C—H⋯π inter­actions (C43—H43⋯*Cg*2^ii^ and C68—H68⋯*Cg*3^iii^; *Cg*2 and *Cg*3 are the centroids of the C54–C59 and C36–C41 rings, respectively; symmetry codes as in Table 1 ▸) (Fig. 4 ▸). The sheets parallel to (010) are cross-linked *via* these C—H⋯π inter­actions into a three-dimensional network.

## Database survey   

A search of the Cambridge Structural Database (CSD, Version 5.40, update February 2019; Groom *et al.*, 2016 ▸) of the compound containing T3PyA yielded only one hit (AXUBIG; Wang *et al.*, 2016 ▸), a trinuclear palladium(II) complex with the exo-tridentate ligand bridging three palladium(II) atoms, namely, μ_3_-tris­[4-(pyridin-3-yl)phen­yl]amine-*N*,*N′*,*N′′*-tris{[1,3-bis­(2,6-diiso­propyl­phen­yl)-2,3-di­hydro-1*H*-imidazol-2-yl­idene]di­chloro­palladium(II)} ethyl acetate solvate trihydrate. A search for compounds containing tris­[4-(pyridin-4-yl)phen­yl]amine (T4PyA), pseudo *D*_3_-symmetric structural isomers of T3PyA, gave 51 hits (48 compounds), including 46 hits for metal complexes (nine, twelve, eleven, seven, three, three and one hits for Zn, Co, Cd, Cu, Ni, Ag and Mn complexes, respectively). Focusing on the coordination number of T4PyA, it featured in 20 hits for the *exo*-tridentate ligand, 24 hits for the *exo*-bidentate ligand, one hit for the monodentate ligand and another hit containing both the *exo*-bidentate and the monodentate ligand. A search for the Zn(dbm)_2_ moiety gave 34 hits (32 compounds). Limiting the search for a pyridine-coordinated Zn(dbm)_2_ moiety gave 15 hits. Seven of these compounds are bipyridyl-ligand-bridged polymeric structures, for example, *catena*-bis­(3-oxo-1,3-di­phenyl­prop-1-enolato)-(μ_2_-4,4′-bipyrid­yl)zinc(II) (AQIQIA; Soldatov *et al.*, 2003 ▸). In this complex, the Zn^II^ atom is ligated by the two N atoms of the 4,4′-bipyridyl ligand and the four O atoms of two *β*-diketonate anions, hence the Zn^II^ atom is *trans*-N_2_O_4_ six-coordinate, similar to that in the title compound.

## Synthesis and crystallization   

T3PyA was prepared by a modification of the reported Suzuki–Miyaura reaction of pyridine boronic esters (Billingsley & Buchwald, 2007 ▸). 3-(4,4,5,5-Tetra­methyl-1,3,2-dioxa­borolan-2-yl)pyridine (820 mg, 4.0 mmol), tris­(4-iodo­phen­yl)amine (623 mg, 1.0 mmol), tetra­kis­(tri­phenyl­phosphine)palladium(0) (23 mg, 0.02 mmol), K_3_PO_4_ (freshly ground, 1.27 g, 6.0 mmol) and 1-butanol (7.5 ml) were placed in a 30 mL round-bottom flask. After the solution was purged with nitro­gen for 15 min, it was heated at 398 K under nitro­gen for 48 h. The solvent was removed under vacuum and the residue was redissolved in ethyl acetate. The organic layer was washed three times with water. The organic layer was then dried over Na_2_SO_4_ and the solvent evaporated to yield a pale-white crude product. The crude product was purified by column chromatography on silica gel [EtOAc/MeOH = 10/1 (*v*/*v*) as eluent] to yield the pure product as a white solid (375 mg, 0.79 mmol, 79%). Zn(dbm)_2_ was prepared according to literature methods (Soldatov *et al.*, 2001 ▸). Single crystals of {[Zn(dbm)_2_(T3PyA)]·THF}_*n*_ were grown by slow evaporation from a THF solution, prepared by filtering a dispersion containing 32 mg of T3PyA and 40 mg of Zn(dbm)_2_ in 12 ml of THF. Colourless crystals suitable for X-ray diffraction were obtained after 2–3 weeks.

## Refinement   

Crystal data, data collection and structure refinement details are summarized in Table 2 ▸. Hydrogen atoms were placed in geometrically calculated positions (C—H = 0.93–0.99 Å) and refined as part of a riding model with *U*_iso_(H) = 1.2*U*_eq_ (C). The solvate THF mol­ecule is disordered over two sets of sites with refined occupancies of 0.631 (7) and 0.369 (7). *EADP* constraints and *SAME* restraints were used to model this disordered mol­ecule. A small number of reflections affected by the beam stop and one outlier (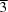
11) were omitted from the refinement.

## Supplementary Material

Crystal structure: contains datablock(s) global, I. DOI: 10.1107/S2056989019012350/is5520sup1.cif

Structure factors: contains datablock(s) I. DOI: 10.1107/S2056989019012350/is5520Isup2.hkl

CCDC reference: 1951564

Additional supporting information:  crystallographic information; 3D view; checkCIF report

## Figures and Tables

**Table 1:** Hydrogen-bond geometry (Å, °) *Cg*1, *Cg*2, *Cg*3 and *Cg*4 are the centroids of the N10/C65–C69, C54–C59, C36–C41 and N11/C70–C74 rings, respectively.

*D*—H⋯*A*	*D*—H	H⋯*A*	*D*⋯*A*	*D*—H⋯*A*
C13—H13⋯O7*A*	0.95	2.47	3.197 (7)	134
C40—H40⋯*Cg*1^i^	0.95	2.74	3.594 (3)	150
C43—H43⋯*Cg*2^ii^	0.95	2.78	3.572 (3)	142
C68—H68⋯*Cg*3^iii^	0.95	2.65	3.513 (3)	152
C75*B*—H75*C*⋯*Cg*4^ii^	0.99	2.78	3.649 (17)	146

Symmetry codes: (i) 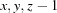; (ii) 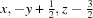; (iii) 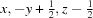.

**Table 2:** Experimental details

Crystal data	
Chemical formula	[Zn(C_15_H_11_O_2_)_2_(C_33_H_24_N_4_)]·C_4_H_8_O
*M*_r_	1060.53
Crystal system, space group	Monoclinic, *P*2_1_/*c*
Temperature (K)	193
*a*, *b*, *c* (Å)	27.2823 (14), 19.7693 (12), 9.9674 (5)
β (°)	94.614 (7)
*V* (Å^3^)	5358.5 (5)
*Z*	4
Radiation type	Mo *K*α
μ (mm^−1^)	0.52
Crystal size (mm)	0.20 × 0.20 × 0.10

Data collection	
Diffractometer	Rigaku R-AXIS RAPID
Absorption correction	Multi-scan (*ABSCOR*; Higashi, 1995 ▸)
*T*_min_, *T*_max_	0.669, 0.950
No. of measured, independent and observed [*F* ^2^ > 2.0σ(*F* ^2^)] reflections	51154, 12244, 8421
*R*_int_	0.083
(sin θ/λ)_max_ (Å^−1^)	0.649

Refinement	
*R*[*F* ^2^ > 2σ(*F* ^2^)], *wR*(*F* ^2^), *S*	0.058, 0.128, 1.04
No. of reflections	12244
No. of parameters	713
No. of restraints	10
H-atom treatment	H-atom parameters constrained
Δρ_max_, Δρ_min_ (e Å^−3^)	0.51, −0.55

Computer programs: *RAPID-AUTO* (Rigaku, 2006 ▸), *SIR92* (Altomare *et al.*, 1993 ▸), *SHELXL2014* (Sheldrick, 2015 ▸), *PLATON* (Spek, 2009 ▸) and *CrystalStructure* (Rigaku, 2016 ▸).

## supplementary crystallographic information

### Crystal data

**Table:**

[Zn(C_15_H_11_O_2_)_2_(C_33_H_24_N_4_)]·C_4_H_8_O	*F*(000) = 2216.00
*M**_r_* = 1060.53	*D*_x_ = 1.315 Mg m^−^^3^
Monoclinic, *P*2_1_/*c*	Mo *K*α radiation, λ = 0.71075 Å
*a* = 27.2823 (14) Å	Cell parameters from 32853 reflections
*b* = 19.7693 (12) Å	θ = 3.0–27.5°
*c* = 9.9674 (5) Å	µ = 0.52 mm^−^^1^
β = 94.614 (7)°	*T* = 193 K
*V* = 5358.5 (5) Å^3^	Block, colorless
*Z* = 4	0.20 × 0.20 × 0.10 mm

### Data collection

**Table:**

Rigaku R-AXIS RAPID diffractometer	8421 reflections with *F*^2^ > 2.0σ(*F*^2^)
Detector resolution: 10.000 pixels mm^-1^	*R*_int_ = 0.083
ω scans	θ_max_ = 27.5°, θ_min_ = 3.0°
Absorption correction: multi-scan (ABSCOR; Higashi, 1995)	*h* = −35→33
*T*_min_ = 0.669, *T*_max_ = 0.950	*k* = −25→25
51154 measured reflections	*l* = −12→12
12244 independent reflections	

### Refinement

**Table:**

Refinement on *F*^2^	Secondary atom site location: difference Fourier map
*R*[*F*^2^ > 2σ(*F*^2^)] = 0.058	Hydrogen site location: inferred from neighbouring sites
*wR*(*F*^2^) = 0.128	H-atom parameters constrained
*S* = 1.04	*w* = 1/[σ^2^(*F*_o_^2^) + (0.0403*P*)^2^ + 4.506*P*] where *P* = (*F*_o_^2^ + 2*F*_c_^2^)/3
12244 reflections	(Δ/σ)_max_ < 0.001
713 parameters	Δρ_max_ = 0.51 e Å^−^^3^
10 restraints	Δρ_min_ = −0.55 e Å^−^^3^
Primary atom site location: structure-invariant direct methods	

### Special details

**Table:**

Geometry. All esds (except the esd in the dihedral angle between two l.s. planes) are estimated using the full covariance matrix. The cell esds are taken into account individually in the estimation of esds in distances, angles and torsion angles; correlations between esds in cell parameters are only used when they are defined by crystal symmetry. An approximate (isotropic) treatment of cell esds is used for estimating esds involving l.s. planes.
Refinement. Refinement was performed using all reflections. The weighted R-factor (wR) and goodness of fit (S) are based on F^2^. R-factor (gt) are based on F. The threshold expression of F^2^ > 2.0 sigma(F^2^) is used only for calculating R-factor (gt).

### Fractional atomic coordinates and isotropic or equivalent isotropic displacement parameters (Å^2^)

**Table:**

	*x*	*y*	*z*	*U*_iso_*/*U*_eq_	Occ. (<1)
Zn1	0.5000	0.5000	1.0000	0.02737 (11)	
Zn2	0.0000	0.5000	0.5000	0.02745 (11)	
O3	0.46823 (7)	0.41755 (9)	0.90263 (18)	0.0343 (4)	
O4	0.45865 (7)	0.56001 (9)	0.86625 (17)	0.0307 (4)	
O5	0.06591 (7)	0.50291 (10)	0.41611 (18)	0.0329 (4)	
O6	−0.03477 (7)	0.53567 (9)	0.32255 (18)	0.0304 (4)	
N8	0.21836 (8)	0.75706 (11)	1.0755 (2)	0.0303 (5)	
N9	0.43981 (8)	0.50191 (11)	1.1341 (2)	0.0302 (5)	
N10	0.00979 (8)	0.60638 (11)	0.5743 (2)	0.0308 (5)	
N11	0.24217 (12)	1.13638 (14)	1.3238 (3)	0.0608 (9)	
C12	0.40705 (12)	0.33603 (15)	0.6141 (3)	0.0412 (7)	
H12	0.3861	0.3717	0.5807	0.049*	
C13	0.40264 (14)	0.27253 (17)	0.5561 (3)	0.0517 (9)	
H13	0.3790	0.2649	0.4824	0.062*	
C14	0.43237 (13)	0.22048 (16)	0.6048 (4)	0.0535 (9)	
H14	0.4299	0.1772	0.5634	0.064*	
C15	0.46578 (13)	0.23118 (16)	0.7136 (4)	0.0526 (9)	
H15	0.4858	0.1949	0.7487	0.063*	
C16	0.47034 (11)	0.29448 (15)	0.7719 (3)	0.0413 (7)	
H16	0.4932	0.3014	0.8477	0.050*	
C17	0.44182 (10)	0.34795 (13)	0.7208 (3)	0.0310 (6)	
C18	0.45079 (10)	0.41676 (14)	0.7820 (3)	0.0304 (6)	
C19	0.44048 (10)	0.47457 (14)	0.7012 (3)	0.0314 (6)	
H19	0.4296	0.4675	0.6093	0.038*	
C20	0.44503 (9)	0.54118 (13)	0.7465 (3)	0.0274 (6)	
C21	0.43292 (9)	0.59851 (13)	0.6508 (3)	0.0264 (6)	
C22	0.41197 (10)	0.65671 (14)	0.6995 (3)	0.0328 (6)	
H22	0.4040	0.6584	0.7905	0.039*	
C23	0.40258 (12)	0.71197 (15)	0.6173 (3)	0.0441 (8)	
H23	0.3871	0.7509	0.6505	0.053*	
C24	0.41568 (14)	0.71056 (18)	0.4867 (4)	0.0576 (10)	
H24	0.4101	0.7491	0.4306	0.069*	
C25	0.43677 (14)	0.65346 (18)	0.4373 (3)	0.0541 (9)	
H25	0.4457	0.6528	0.3471	0.065*	
C26	0.44509 (11)	0.59711 (16)	0.5176 (3)	0.0384 (7)	
H26	0.4591	0.5575	0.4823	0.046*	
C27	0.13575 (10)	0.51169 (13)	0.1293 (3)	0.0322 (6)	
H27	0.1099	0.5097	0.0597	0.039*	
C28	0.18406 (11)	0.50517 (15)	0.0978 (3)	0.0389 (7)	
H28	0.1911	0.4978	0.0072	0.047*	
C29	0.22161 (11)	0.50935 (16)	0.1968 (4)	0.0449 (8)	
H29	0.2547	0.5051	0.1749	0.054*	
C30	0.21144 (11)	0.51977 (18)	0.3292 (4)	0.0480 (8)	
H30	0.2376	0.5236	0.3978	0.058*	
C31	0.16317 (10)	0.52457 (15)	0.3615 (3)	0.0374 (7)	
H31	0.1563	0.5303	0.4527	0.045*	
C32	0.12475 (10)	0.52116 (13)	0.2621 (3)	0.0284 (6)	
C33	0.07303 (10)	0.52595 (13)	0.3015 (3)	0.0279 (6)	
C34	0.03721 (10)	0.55579 (14)	0.2102 (3)	0.0304 (6)	
H34	0.0486	0.5761	0.1320	0.037*	
C35	−0.01330 (9)	0.55847 (13)	0.2236 (3)	0.0263 (6)	
C36	−0.04605 (10)	0.59145 (13)	0.1145 (3)	0.0283 (6)	
C37	−0.09199 (10)	0.61582 (15)	0.1451 (3)	0.0346 (6)	
H37	−0.1020	0.6103	0.2336	0.042*	
C38	−0.12305 (11)	0.64781 (15)	0.0484 (3)	0.0412 (7)	
H38	−0.1540	0.6643	0.0711	0.049*	
C39	−0.10916 (11)	0.65585 (16)	−0.0810 (3)	0.0439 (8)	
H39	−0.1302	0.6784	−0.1470	0.053*	
C40	−0.06450 (12)	0.63083 (16)	−0.1134 (3)	0.0420 (7)	
H40	−0.0551	0.6353	−0.2027	0.050*	
C41	−0.03314 (10)	0.59917 (14)	−0.0170 (3)	0.0339 (6)	
H41	−0.0024	0.5825	−0.0408	0.041*	
C42	0.25467 (9)	0.70554 (13)	1.1048 (3)	0.0277 (6)	
C43	0.27359 (10)	0.66926 (14)	1.0018 (3)	0.0308 (6)	
H43	0.2615	0.6770	0.9111	0.037*	
C44	0.31018 (10)	0.62179 (14)	1.0312 (3)	0.0301 (6)	
H44	0.3236	0.5981	0.9597	0.036*	
C45	0.32775 (9)	0.60808 (13)	1.1632 (3)	0.0268 (6)	
C46	0.30764 (10)	0.64398 (14)	1.2660 (3)	0.0310 (6)	
H46	0.3188	0.6353	1.3571	0.037*	
C47	0.27148 (10)	0.69210 (14)	1.2365 (3)	0.0301 (6)	
H47	0.2581	0.7161	1.3076	0.036*	
C48	0.17551 (9)	0.74194 (13)	0.9924 (3)	0.0258 (5)	
C49	0.15424 (10)	0.78918 (13)	0.9014 (3)	0.0299 (6)	
H49	0.1680	0.8332	0.8976	0.036*	
C50	0.11354 (10)	0.77273 (13)	0.8170 (3)	0.0307 (6)	
H50	0.0997	0.8058	0.7559	0.037*	
C51	0.09207 (9)	0.70849 (13)	0.8189 (3)	0.0266 (6)	
C52	0.11303 (9)	0.66233 (13)	0.9123 (3)	0.0288 (6)	
H52	0.0990	0.6185	0.9170	0.035*	
C53	0.15362 (10)	0.67833 (13)	0.9983 (3)	0.0286 (6)	
H53	0.1667	0.6459	1.0617	0.034*	
C54	0.22980 (9)	0.82375 (13)	1.1193 (3)	0.0267 (6)	
C55	0.27766 (9)	0.84818 (13)	1.1208 (3)	0.0287 (6)	
H55	0.3027	0.8203	1.0893	0.034*	
C56	0.28929 (10)	0.91228 (14)	1.1673 (3)	0.0300 (6)	
H56	0.3222	0.9280	1.1662	0.036*	
C57	0.25386 (10)	0.95476 (13)	1.2162 (3)	0.0281 (6)	
C58	0.20597 (10)	0.92973 (13)	1.2127 (3)	0.0302 (6)	
H58	0.1809	0.9576	1.2439	0.036*	
C59	0.19376 (10)	0.86593 (14)	1.1655 (3)	0.0295 (6)	
H59	0.1607	0.8506	1.1643	0.035*	
C60	0.36486 (9)	0.55430 (13)	1.1920 (3)	0.0265 (6)	
C61	0.40591 (10)	0.55061 (13)	1.1191 (3)	0.0290 (6)	
H61	0.4102	0.5849	1.0544	0.035*	
C62	0.43328 (11)	0.45340 (14)	1.2257 (3)	0.0338 (6)	
H62	0.4569	0.4181	1.2373	0.041*	
C63	0.39405 (11)	0.45289 (15)	1.3029 (3)	0.0381 (7)	
H63	0.3905	0.4177	1.3663	0.046*	
C64	0.35945 (10)	0.50436 (15)	1.2875 (3)	0.0346 (6)	
H64	0.3324	0.5054	1.3418	0.041*	
C65	0.05086 (10)	0.69018 (13)	0.7199 (3)	0.0294 (6)	
C66	0.04572 (9)	0.62529 (13)	0.6664 (3)	0.0289 (6)	
H66	0.0692	0.5922	0.6972	0.035*	
C67	−0.02290 (11)	0.65327 (15)	0.5312 (3)	0.0383 (7)	
H67	−0.0488	0.6408	0.4665	0.046*	
C68	−0.02076 (12)	0.71905 (16)	0.5767 (3)	0.0450 (8)	
H68	−0.0444	0.7512	0.5424	0.054*	
C69	0.01594 (11)	0.73772 (15)	0.6722 (3)	0.0392 (7)	
H69	0.0175	0.7828	0.7056	0.047*	
C70	0.26721 (11)	1.02246 (14)	1.2723 (3)	0.0339 (6)	
C71	0.23316 (13)	1.07453 (16)	1.2725 (3)	0.0464 (8)	
H71	0.2009	1.0657	1.2332	0.056*	
C72	0.28771 (15)	1.14791 (19)	1.3772 (4)	0.0627 (11)	
H72	0.2950	1.1912	1.4152	0.075*	
C73	0.32479 (14)	1.10082 (18)	1.3806 (4)	0.0545 (9)	
H73	0.3569	1.1117	1.4184	0.065*	
C74	0.31441 (12)	1.03756 (16)	1.3278 (3)	0.0418 (7)	
H74	0.3395	1.0042	1.3294	0.050*	
O7A	0.30876 (19)	0.3309 (4)	0.3809 (5)	0.0718 (15)	0.631 (7)
C75A	0.2656 (4)	0.3526 (8)	0.4433 (8)	0.071 (2)	0.631 (7)
H75A	0.2644	0.4026	0.4491	0.085*	0.631 (7)
H75B	0.2651	0.3335	0.5350	0.085*	0.631 (7)
C76A	0.2224 (4)	0.3257 (8)	0.3513 (9)	0.071 (2)	0.631 (7)
H76A	0.2103	0.2824	0.3859	0.085*	0.631 (7)
H76B	0.1949	0.3587	0.3443	0.085*	0.631 (7)
C77A	0.2426 (3)	0.3159 (7)	0.2184 (9)	0.064 (2)	0.631 (7)
H77A	0.2273	0.3479	0.1511	0.076*	0.631 (7)
H77B	0.2366	0.2692	0.1854	0.076*	0.631 (7)
C78A	0.2966 (4)	0.3293 (12)	0.2427 (12)	0.072 (3)	0.631 (7)
H78A	0.3154	0.2932	0.2010	0.087*	0.631 (7)
H78B	0.3049	0.3731	0.2020	0.087*	0.631 (7)
O7B	0.3004 (4)	0.3737 (6)	0.3829 (10)	0.0718 (15)	0.369 (7)
C75B	0.2723 (7)	0.3464 (15)	0.4837 (14)	0.071 (2)	0.369 (7)
H75C	0.2667	0.3807	0.5533	0.085*	0.369 (7)
H75D	0.2890	0.3067	0.5274	0.085*	0.369 (7)
C76B	0.2240 (7)	0.3260 (16)	0.4069 (17)	0.071 (2)	0.369 (7)
H76C	0.2128	0.2816	0.4389	0.085*	0.369 (7)
H76D	0.1982	0.3601	0.4196	0.085*	0.369 (7)
C77B	0.2343 (7)	0.3219 (14)	0.2619 (18)	0.064 (2)	0.369 (7)
H77C	0.2140	0.3549	0.2071	0.076*	0.369 (7)
H77D	0.2276	0.2759	0.2257	0.076*	0.369 (7)
C78B	0.2879 (8)	0.339 (2)	0.263 (2)	0.072 (3)	0.369 (7)
H78C	0.3077	0.2970	0.2592	0.087*	0.369 (7)
H78D	0.2940	0.3674	0.1845	0.087*	0.369 (7)

### Atomic displacement parameters (Å^2^)

**Table:**

	*U*^11^	*U*^22^	*U*^33^	*U*^12^	*U*^13^	*U*^23^
Zn1	0.0355 (2)	0.0210 (2)	0.0252 (2)	0.00777 (19)	−0.00072 (18)	−0.00111 (18)
Zn2	0.0287 (2)	0.0261 (2)	0.0277 (2)	−0.00193 (19)	0.00256 (18)	0.00102 (19)
O3	0.0497 (12)	0.0221 (10)	0.0298 (10)	0.0016 (9)	−0.0045 (9)	0.0008 (8)
O4	0.0418 (11)	0.0241 (10)	0.0253 (9)	0.0101 (8)	−0.0045 (8)	−0.0023 (8)
O5	0.0307 (9)	0.0352 (11)	0.0329 (10)	−0.0002 (9)	0.0039 (8)	0.0035 (9)
O6	0.0299 (10)	0.0306 (11)	0.0306 (10)	−0.0008 (8)	0.0020 (8)	0.0031 (8)
N8	0.0307 (12)	0.0192 (11)	0.0399 (13)	0.0023 (9)	−0.0033 (10)	−0.0039 (10)
N9	0.0360 (12)	0.0222 (11)	0.0322 (11)	0.0076 (10)	0.0006 (10)	−0.0010 (10)
N10	0.0322 (12)	0.0273 (12)	0.0323 (12)	−0.0003 (10)	−0.0010 (10)	−0.0013 (10)
N11	0.067 (2)	0.0284 (15)	0.090 (2)	−0.0033 (14)	0.0235 (18)	−0.0145 (15)
C12	0.0544 (19)	0.0268 (16)	0.0416 (17)	−0.0041 (14)	−0.0021 (15)	0.0012 (13)
C13	0.073 (2)	0.0350 (18)	0.0444 (18)	−0.0114 (17)	−0.0111 (18)	−0.0035 (15)
C14	0.071 (2)	0.0231 (16)	0.066 (2)	−0.0053 (16)	0.004 (2)	−0.0109 (16)
C15	0.053 (2)	0.0221 (16)	0.081 (3)	0.0039 (14)	−0.0039 (19)	−0.0039 (16)
C16	0.0415 (17)	0.0243 (15)	0.0567 (19)	−0.0017 (13)	−0.0043 (15)	−0.0005 (14)
C17	0.0374 (15)	0.0202 (13)	0.0356 (15)	−0.0025 (11)	0.0043 (13)	0.0009 (11)
C18	0.0338 (14)	0.0231 (14)	0.0342 (15)	0.0035 (11)	0.0025 (12)	−0.0015 (11)
C19	0.0411 (16)	0.0254 (14)	0.0266 (13)	0.0020 (12)	−0.0038 (12)	0.0004 (11)
C20	0.0274 (13)	0.0245 (14)	0.0305 (14)	0.0025 (11)	0.0034 (12)	0.0017 (11)
C21	0.0262 (13)	0.0228 (13)	0.0295 (13)	−0.0024 (11)	−0.0016 (11)	0.0023 (11)
C22	0.0366 (15)	0.0253 (15)	0.0355 (15)	0.0009 (12)	−0.0028 (13)	0.0019 (12)
C23	0.0529 (19)	0.0234 (16)	0.053 (2)	0.0018 (14)	−0.0120 (16)	0.0046 (14)
C24	0.081 (3)	0.0334 (19)	0.055 (2)	−0.0125 (18)	−0.0185 (19)	0.0205 (16)
C25	0.081 (3)	0.049 (2)	0.0326 (16)	−0.0166 (19)	0.0001 (17)	0.0128 (15)
C26	0.0490 (18)	0.0347 (17)	0.0319 (15)	−0.0063 (14)	0.0062 (14)	0.0018 (13)
C27	0.0342 (14)	0.0247 (15)	0.0379 (15)	0.0009 (11)	0.0036 (13)	0.0044 (11)
C28	0.0400 (16)	0.0296 (16)	0.0489 (17)	−0.0017 (13)	0.0153 (14)	0.0032 (14)
C29	0.0298 (15)	0.0380 (19)	0.068 (2)	−0.0031 (13)	0.0109 (15)	0.0005 (16)
C30	0.0321 (16)	0.055 (2)	0.056 (2)	−0.0032 (14)	−0.0022 (15)	−0.0050 (16)
C31	0.0328 (15)	0.0380 (16)	0.0413 (16)	−0.0035 (13)	0.0032 (13)	−0.0050 (13)
C32	0.0307 (14)	0.0156 (12)	0.0391 (15)	−0.0025 (10)	0.0049 (12)	−0.0001 (11)
C33	0.0295 (13)	0.0178 (12)	0.0368 (15)	−0.0039 (11)	0.0051 (12)	−0.0026 (11)
C34	0.0327 (14)	0.0256 (14)	0.0334 (14)	−0.0008 (11)	0.0054 (12)	0.0033 (12)
C35	0.0320 (14)	0.0167 (13)	0.0300 (13)	−0.0020 (11)	0.0006 (12)	−0.0043 (10)
C36	0.0295 (14)	0.0232 (14)	0.0318 (14)	−0.0053 (11)	0.0012 (12)	−0.0001 (11)
C37	0.0314 (14)	0.0339 (16)	0.0383 (16)	−0.0019 (12)	0.0017 (13)	−0.0002 (13)
C38	0.0332 (15)	0.0336 (17)	0.0556 (19)	0.0014 (13)	−0.0032 (14)	0.0022 (15)
C39	0.0430 (18)	0.0367 (18)	0.0492 (19)	−0.0061 (14)	−0.0122 (15)	0.0082 (15)
C40	0.0480 (18)	0.0399 (18)	0.0367 (16)	−0.0104 (15)	−0.0043 (14)	0.0057 (14)
C41	0.0343 (15)	0.0317 (16)	0.0355 (15)	−0.0058 (12)	0.0014 (13)	−0.0025 (12)
C42	0.0265 (13)	0.0207 (13)	0.0361 (14)	0.0010 (11)	0.0034 (12)	−0.0017 (11)
C43	0.0388 (15)	0.0255 (14)	0.0277 (13)	0.0045 (12)	0.0005 (12)	0.0008 (11)
C44	0.0340 (14)	0.0279 (14)	0.0287 (14)	0.0034 (12)	0.0051 (12)	−0.0032 (11)
C45	0.0279 (13)	0.0227 (13)	0.0304 (14)	0.0008 (11)	0.0056 (11)	0.0013 (11)
C46	0.0369 (15)	0.0292 (15)	0.0271 (14)	0.0040 (12)	0.0038 (12)	0.0019 (11)
C47	0.0337 (14)	0.0272 (15)	0.0299 (14)	0.0033 (12)	0.0064 (12)	−0.0047 (11)
C48	0.0262 (13)	0.0212 (13)	0.0302 (13)	0.0013 (10)	0.0030 (11)	−0.0031 (11)
C49	0.0310 (14)	0.0212 (14)	0.0374 (15)	−0.0029 (11)	0.0015 (12)	−0.0013 (11)
C50	0.0348 (15)	0.0221 (14)	0.0353 (15)	0.0035 (11)	0.0033 (12)	0.0022 (11)
C51	0.0292 (13)	0.0200 (13)	0.0310 (14)	0.0008 (11)	0.0040 (12)	−0.0025 (11)
C52	0.0314 (14)	0.0201 (13)	0.0353 (14)	−0.0024 (11)	0.0049 (12)	−0.0002 (11)
C53	0.0325 (14)	0.0215 (13)	0.0316 (14)	0.0030 (11)	0.0020 (12)	0.0006 (11)
C54	0.0301 (13)	0.0211 (13)	0.0286 (13)	0.0002 (11)	0.0007 (11)	−0.0005 (11)
C55	0.0285 (13)	0.0243 (14)	0.0337 (14)	0.0040 (11)	0.0046 (12)	−0.0004 (11)
C56	0.0280 (13)	0.0277 (14)	0.0341 (14)	−0.0045 (11)	0.0022 (12)	0.0050 (12)
C57	0.0334 (14)	0.0225 (13)	0.0284 (13)	−0.0005 (11)	0.0026 (12)	0.0011 (11)
C58	0.0323 (14)	0.0246 (14)	0.0339 (14)	0.0021 (11)	0.0050 (12)	−0.0044 (11)
C59	0.0268 (13)	0.0284 (15)	0.0337 (14)	−0.0006 (11)	0.0053 (12)	−0.0018 (12)
C60	0.0313 (14)	0.0205 (13)	0.0275 (13)	0.0018 (11)	0.0016 (11)	−0.0017 (11)
C61	0.0376 (15)	0.0209 (13)	0.0284 (13)	0.0053 (11)	0.0022 (12)	0.0022 (11)
C62	0.0401 (16)	0.0202 (14)	0.0405 (16)	0.0057 (12)	0.0001 (14)	0.0024 (12)
C63	0.0456 (17)	0.0266 (15)	0.0420 (16)	0.0023 (13)	0.0023 (14)	0.0117 (13)
C64	0.0341 (14)	0.0344 (16)	0.0357 (14)	0.0010 (13)	0.0059 (12)	0.0054 (13)
C65	0.0300 (14)	0.0244 (14)	0.0337 (14)	−0.0002 (11)	0.0034 (12)	0.0008 (11)
C66	0.0272 (13)	0.0219 (13)	0.0373 (15)	0.0023 (11)	−0.0001 (12)	−0.0001 (11)
C67	0.0368 (16)	0.0352 (17)	0.0406 (16)	0.0012 (13)	−0.0108 (13)	0.0003 (13)
C68	0.0470 (18)	0.0313 (17)	0.0537 (19)	0.0099 (14)	−0.0152 (16)	−0.0001 (14)
C69	0.0416 (16)	0.0251 (15)	0.0491 (18)	0.0034 (13)	−0.0075 (14)	−0.0033 (13)
C70	0.0418 (16)	0.0258 (14)	0.0351 (15)	−0.0057 (12)	0.0084 (13)	−0.0010 (12)
C71	0.0488 (18)	0.0295 (17)	0.062 (2)	−0.0036 (14)	0.0134 (17)	−0.0065 (15)
C72	0.076 (3)	0.037 (2)	0.079 (3)	−0.0200 (19)	0.024 (2)	−0.0221 (19)
C73	0.058 (2)	0.044 (2)	0.062 (2)	−0.0194 (17)	0.0076 (18)	−0.0151 (17)
C74	0.0472 (18)	0.0316 (17)	0.0469 (18)	−0.0088 (14)	0.0066 (15)	−0.0037 (14)
O7A	0.065 (2)	0.092 (5)	0.057 (2)	−0.002 (3)	−0.0053 (19)	0.006 (3)
C75A	0.098 (5)	0.070 (4)	0.045 (5)	0.005 (4)	0.003 (5)	0.015 (5)
C76A	0.072 (3)	0.078 (4)	0.066 (8)	−0.009 (3)	0.023 (5)	−0.012 (8)
C77A	0.064 (5)	0.057 (4)	0.069 (7)	0.002 (3)	0.005 (4)	−0.013 (5)
C78A	0.070 (5)	0.080 (8)	0.067 (5)	0.015 (6)	0.007 (3)	−0.005 (4)
O7B	0.065 (2)	0.092 (5)	0.057 (2)	−0.002 (3)	−0.0053 (19)	0.006 (3)
C75B	0.098 (5)	0.070 (4)	0.045 (5)	0.005 (4)	0.003 (5)	0.015 (5)
C76B	0.072 (3)	0.078 (4)	0.066 (8)	−0.009 (3)	0.023 (5)	−0.012 (8)
C77B	0.064 (5)	0.057 (4)	0.069 (7)	0.002 (3)	0.005 (4)	−0.013 (5)
C78B	0.070 (5)	0.080 (8)	0.067 (5)	0.015 (6)	0.007 (3)	−0.005 (4)

### Geometric parameters (Å, º)

**Table:**

Zn1—O3^i^	2.0528 (19)	C43—C44	1.384 (4)
Zn1—O3	2.0529 (19)	C43—H43	0.9500
Zn1—O4	2.0531 (18)	C44—C45	1.390 (4)
Zn1—O4^i^	2.0531 (18)	C44—H44	0.9500
Zn1—N9^i^	2.199 (2)	C45—C46	1.395 (3)
Zn1—N9	2.199 (2)	C45—C60	1.480 (4)
Zn2—O5	2.0440 (17)	C46—C47	1.385 (4)
Zn2—O5^ii^	2.0440 (17)	C46—H46	0.9500
Zn2—O6^ii^	2.0628 (18)	C47—H47	0.9500
Zn2—O6	2.0629 (18)	C48—C53	1.395 (4)
Zn2—N10	2.238 (2)	C48—C49	1.396 (4)
Zn2—N10^ii^	2.238 (2)	C49—C50	1.377 (4)
O3—C18	1.257 (3)	C49—H49	0.9500
O4—C20	1.276 (3)	C50—C51	1.399 (4)
O5—C33	1.260 (3)	C50—H50	0.9500
O6—C35	1.270 (3)	C51—C52	1.393 (4)
N8—C48	1.409 (3)	C51—C65	1.480 (4)
N8—C54	1.416 (3)	C52—C53	1.381 (4)
N8—C42	1.434 (3)	C52—H52	0.9500
N9—C61	1.335 (3)	C53—H53	0.9500
N9—C62	1.346 (3)	C54—C55	1.391 (4)
N10—C67	1.333 (4)	C54—C59	1.395 (4)
N10—C66	1.341 (3)	C55—C56	1.378 (4)
N11—C72	1.331 (5)	C55—H55	0.9500
N11—C71	1.340 (4)	C56—C57	1.397 (4)
C12—C13	1.383 (4)	C56—H56	0.9500
C12—C17	1.387 (4)	C57—C58	1.395 (4)
C12—H12	0.9500	C57—C70	1.485 (4)
C13—C14	1.374 (5)	C58—C59	1.378 (4)
C13—H13	0.9500	C58—H58	0.9500
C14—C15	1.376 (5)	C59—H59	0.9500
C14—H14	0.9500	C60—C61	1.385 (4)
C15—C16	1.381 (4)	C60—C64	1.388 (4)
C15—H15	0.9500	C61—H61	0.9500
C16—C17	1.385 (4)	C62—C63	1.367 (4)
C16—H16	0.9500	C62—H62	0.9500
C17—C18	1.503 (4)	C63—C64	1.388 (4)
C18—C19	1.413 (4)	C63—H63	0.9500
C19—C20	1.395 (4)	C64—H64	0.9500
C19—H19	0.9500	C65—C66	1.392 (4)
C20—C21	1.501 (4)	C65—C69	1.394 (4)
C21—C22	1.390 (4)	C66—H66	0.9500
C21—C26	1.395 (4)	C67—C68	1.377 (4)
C22—C23	1.377 (4)	C67—H67	0.9500
C22—H22	0.9500	C68—C69	1.376 (4)
C23—C24	1.377 (5)	C68—H68	0.9500
C23—H23	0.9500	C69—H69	0.9500
C24—C25	1.376 (5)	C70—C71	1.387 (4)
C24—H24	0.9500	C70—C74	1.393 (4)
C25—C26	1.380 (4)	C71—H71	0.9500
C25—H25	0.9500	C72—C73	1.373 (5)
C26—H26	0.9500	C72—H72	0.9500
C27—C28	1.385 (4)	C73—C74	1.377 (4)
C27—C32	1.394 (4)	C73—H73	0.9500
C27—H27	0.9500	C74—H74	0.9500
C28—C29	1.366 (4)	O7A—C78A	1.391 (12)
C28—H28	0.9500	O7A—C75A	1.441 (11)
C29—C30	1.386 (5)	C75A—C76A	1.529 (9)
C29—H29	0.9500	C75A—H75A	0.9900
C30—C31	1.384 (4)	C75A—H75B	0.9900
C30—H30	0.9500	C76A—C77A	1.489 (8)
C31—C32	1.385 (4)	C76A—H76A	0.9900
C31—H31	0.9500	C76A—H76B	0.9900
C32—C33	1.498 (4)	C77A—C78A	1.496 (9)
C33—C34	1.410 (4)	C77A—H77A	0.9900
C34—C35	1.396 (4)	C77A—H77B	0.9900
C34—H34	0.9500	C78A—H78A	0.9900
C35—C36	1.500 (4)	C78A—H78B	0.9900
C36—C41	1.392 (4)	O7B—C78B	1.399 (15)
C36—C37	1.399 (4)	O7B—C75B	1.418 (14)
C37—C38	1.384 (4)	C75B—C76B	1.525 (14)
C37—H37	0.9500	C75B—H75C	0.9900
C38—C39	1.383 (4)	C75B—H75D	0.9900
C38—H38	0.9500	C76B—C77B	1.496 (13)
C39—C40	1.377 (4)	C76B—H76C	0.9900
C39—H39	0.9500	C76B—H76D	0.9900
C40—C41	1.384 (4)	C77B—C78B	1.500 (14)
C40—H40	0.9500	C77B—H77C	0.9900
C41—H41	0.9500	C77B—H77D	0.9900
C42—C47	1.381 (4)	C78B—H78C	0.9900
C42—C43	1.386 (4)	C78B—H78D	0.9900
			
O3^i^—Zn1—O3	180.0	C45—C44—H44	119.3
O3^i^—Zn1—O4	92.14 (8)	C44—C45—C46	118.0 (2)
O3—Zn1—O4	87.87 (8)	C44—C45—C60	120.1 (2)
O3^i^—Zn1—O4^i^	87.86 (8)	C46—C45—C60	121.8 (2)
O3—Zn1—O4^i^	92.14 (8)	C47—C46—C45	120.6 (2)
O4—Zn1—O4^i^	180.0	C47—C46—H46	119.7
O3^i^—Zn1—N9^i^	89.62 (8)	C45—C46—H46	119.7
O3—Zn1—N9^i^	90.38 (8)	C42—C47—C46	120.7 (2)
O4—Zn1—N9^i^	90.77 (8)	C42—C47—H47	119.7
O4^i^—Zn1—N9^i^	89.23 (8)	C46—C47—H47	119.7
O3^i^—Zn1—N9	90.38 (8)	C53—C48—C49	118.2 (2)
O3—Zn1—N9	89.62 (8)	C53—C48—N8	120.3 (2)
O4—Zn1—N9	89.23 (8)	C49—C48—N8	121.5 (2)
O4^i^—Zn1—N9	90.77 (8)	C50—C49—C48	120.8 (2)
N9^i^—Zn1—N9	180.00 (7)	C50—C49—H49	119.6
O5—Zn2—O5^ii^	180.0	C48—C49—H49	119.6
O5—Zn2—O6^ii^	90.08 (7)	C49—C50—C51	121.6 (3)
O5^ii^—Zn2—O6^ii^	89.92 (7)	C49—C50—H50	119.2
O5—Zn2—O6	89.92 (7)	C51—C50—H50	119.2
O5^ii^—Zn2—O6	90.08 (7)	C52—C51—C50	117.0 (2)
O6^ii^—Zn2—O6	180.0	C52—C51—C65	122.3 (2)
O5—Zn2—N10	91.34 (8)	C50—C51—C65	120.6 (2)
O5^ii^—Zn2—N10	88.66 (8)	C53—C52—C51	122.0 (2)
O6^ii^—Zn2—N10	90.18 (8)	C53—C52—H52	119.0
O6—Zn2—N10	89.82 (8)	C51—C52—H52	119.0
O5—Zn2—N10^ii^	88.66 (8)	C52—C53—C48	120.3 (2)
O5^ii^—Zn2—N10^ii^	91.34 (8)	C52—C53—H53	119.8
O6^ii^—Zn2—N10^ii^	89.82 (8)	C48—C53—H53	119.8
O6—Zn2—N10^ii^	90.18 (8)	C55—C54—C59	118.3 (2)
N10—Zn2—N10^ii^	180.0	C55—C54—N8	120.7 (2)
C18—O3—Zn1	125.10 (17)	C59—C54—N8	121.0 (2)
C20—O4—Zn1	122.82 (16)	C56—C55—C54	120.9 (2)
C33—O5—Zn2	125.89 (18)	C56—C55—H55	119.6
C35—O6—Zn2	125.35 (17)	C54—C55—H55	119.6
C48—N8—C54	122.0 (2)	C55—C56—C57	121.5 (2)
C48—N8—C42	119.7 (2)	C55—C56—H56	119.2
C54—N8—C42	117.8 (2)	C57—C56—H56	119.2
C61—N9—C62	117.5 (2)	C58—C57—C56	116.8 (2)
C61—N9—Zn1	119.27 (18)	C58—C57—C70	122.0 (2)
C62—N9—Zn1	123.11 (18)	C56—C57—C70	121.2 (2)
C67—N10—C66	117.4 (2)	C59—C58—C57	122.2 (2)
C67—N10—Zn2	119.21 (19)	C59—C58—H58	118.9
C66—N10—Zn2	123.32 (18)	C57—C58—H58	118.9
C72—N11—C71	116.5 (3)	C58—C59—C54	120.2 (2)
C13—C12—C17	120.4 (3)	C58—C59—H59	119.9
C13—C12—H12	119.8	C54—C59—H59	119.9
C17—C12—H12	119.8	C61—C60—C64	117.2 (2)
C14—C13—C12	120.2 (3)	C61—C60—C45	120.3 (2)
C14—C13—H13	119.9	C64—C60—C45	122.4 (2)
C12—C13—H13	119.9	N9—C61—C60	124.2 (2)
C13—C14—C15	119.9 (3)	N9—C61—H61	117.9
C13—C14—H14	120.1	C60—C61—H61	117.9
C15—C14—H14	120.1	N9—C62—C63	122.7 (3)
C14—C15—C16	120.2 (3)	N9—C62—H62	118.7
C14—C15—H15	119.9	C63—C62—H62	118.7
C16—C15—H15	119.9	C62—C63—C64	119.2 (3)
C15—C16—C17	120.5 (3)	C62—C63—H63	120.4
C15—C16—H16	119.7	C64—C63—H63	120.4
C17—C16—H16	119.7	C60—C64—C63	119.2 (2)
C16—C17—C12	118.8 (3)	C60—C64—H64	120.4
C16—C17—C18	118.2 (3)	C63—C64—H64	120.4
C12—C17—C18	123.0 (3)	C66—C65—C69	116.5 (3)
O3—C18—C19	125.3 (3)	C66—C65—C51	121.9 (2)
O3—C18—C17	115.9 (2)	C69—C65—C51	121.6 (2)
C19—C18—C17	118.8 (2)	N10—C66—C65	124.4 (2)
C20—C19—C18	124.8 (2)	N10—C66—H66	117.8
C20—C19—H19	117.6	C65—C66—H66	117.8
C18—C19—H19	117.6	N10—C67—C68	122.8 (3)
O4—C20—C19	126.1 (2)	N10—C67—H67	118.6
O4—C20—C21	114.0 (2)	C68—C67—H67	118.6
C19—C20—C21	119.9 (2)	C69—C68—C67	119.4 (3)
C22—C21—C26	119.0 (3)	C69—C68—H68	120.3
C22—C21—C20	118.6 (2)	C67—C68—H68	120.3
C26—C21—C20	122.2 (2)	C68—C69—C65	119.6 (3)
C23—C22—C21	120.7 (3)	C68—C69—H69	120.2
C23—C22—H22	119.6	C65—C69—H69	120.2
C21—C22—H22	119.6	C71—C70—C74	116.0 (3)
C22—C23—C24	119.7 (3)	C71—C70—C57	121.7 (3)
C22—C23—H23	120.1	C74—C70—C57	122.3 (3)
C24—C23—H23	120.1	N11—C71—C70	125.1 (3)
C25—C24—C23	120.2 (3)	N11—C71—H71	117.4
C25—C24—H24	119.9	C70—C71—H71	117.4
C23—C24—H24	119.9	N11—C72—C73	123.7 (3)
C24—C25—C26	120.6 (3)	N11—C72—H72	118.1
C24—C25—H25	119.7	C73—C72—H72	118.1
C26—C25—H25	119.7	C72—C73—C74	118.5 (3)
C25—C26—C21	119.7 (3)	C72—C73—H73	120.7
C25—C26—H26	120.1	C74—C73—H73	120.7
C21—C26—H26	120.1	C73—C74—C70	120.1 (3)
C28—C27—C32	120.6 (3)	C73—C74—H74	119.9
C28—C27—H27	119.7	C70—C74—H74	119.9
C32—C27—H27	119.7	C78A—O7A—C75A	107.2 (7)
C29—C28—C27	120.2 (3)	O7A—C75A—C76A	104.8 (8)
C29—C28—H28	119.9	O7A—C75A—H75A	110.8
C27—C28—H28	119.9	C76A—C75A—H75A	110.8
C28—C29—C30	120.0 (3)	O7A—C75A—H75B	110.8
C28—C29—H29	120.0	C76A—C75A—H75B	110.8
C30—C29—H29	120.0	H75A—C75A—H75B	108.9
C31—C30—C29	119.9 (3)	C77A—C76A—C75A	104.7 (6)
C31—C30—H30	120.0	C77A—C76A—H76A	110.8
C29—C30—H30	120.0	C75A—C76A—H76A	110.8
C30—C31—C32	120.7 (3)	C77A—C76A—H76B	110.8
C30—C31—H31	119.7	C75A—C76A—H76B	110.8
C32—C31—H31	119.7	H76A—C76A—H76B	108.9
C31—C32—C27	118.5 (2)	C76A—C77A—C78A	105.3 (7)
C31—C32—C33	119.0 (2)	C76A—C77A—H77A	110.7
C27—C32—C33	122.5 (3)	C78A—C77A—H77A	110.7
O5—C33—C34	125.9 (2)	C76A—C77A—H77B	110.7
O5—C33—C32	115.6 (2)	C78A—C77A—H77B	110.7
C34—C33—C32	118.4 (2)	H77A—C77A—H77B	108.8
C35—C34—C33	126.4 (2)	O7A—C78A—C77A	108.5 (8)
C35—C34—H34	116.8	O7A—C78A—H78A	110.0
C33—C34—H34	116.8	C77A—C78A—H78A	110.0
O6—C35—C34	125.5 (2)	O7A—C78A—H78B	110.0
O6—C35—C36	115.6 (2)	C77A—C78A—H78B	110.0
C34—C35—C36	118.9 (2)	H78A—C78A—H78B	108.4
C41—C36—C37	117.8 (3)	C78B—O7B—C75B	108.0 (16)
C41—C36—C35	123.3 (2)	O7B—C75B—C76B	103.9 (11)
C37—C36—C35	118.9 (2)	O7B—C75B—H75C	111.0
C38—C37—C36	121.0 (3)	C76B—C75B—H75C	111.0
C38—C37—H37	119.5	O7B—C75B—H75D	111.0
C36—C37—H37	119.5	C76B—C75B—H75D	111.0
C39—C38—C37	120.3 (3)	H75C—C75B—H75D	109.0
C39—C38—H38	119.9	C77B—C76B—C75B	106.1 (11)
C37—C38—H38	119.9	C77B—C76B—H76C	110.5
C40—C39—C38	119.4 (3)	C75B—C76B—H76C	110.5
C40—C39—H39	120.3	C77B—C76B—H76D	110.5
C38—C39—H39	120.3	C75B—C76B—H76D	110.5
C39—C40—C41	120.6 (3)	H76C—C76B—H76D	108.7
C39—C40—H40	119.7	C76B—C77B—C78B	103.9 (12)
C41—C40—H40	119.7	C76B—C77B—H77C	111.0
C40—C41—C36	121.0 (3)	C78B—C77B—H77C	111.0
C40—C41—H41	119.5	C76B—C77B—H77D	111.0
C36—C41—H41	119.5	C78B—C77B—H77D	111.0
C47—C42—C43	119.3 (2)	H77C—C77B—H77D	109.0
C47—C42—N8	120.1 (2)	O7B—C78B—C77B	106.7 (12)
C43—C42—N8	120.6 (2)	O7B—C78B—H78C	110.4
C44—C43—C42	120.0 (3)	C77B—C78B—H78C	110.4
C44—C43—H43	120.0	O7B—C78B—H78D	110.4
C42—C43—H43	120.0	C77B—C78B—H78D	110.4
C43—C44—C45	121.4 (2)	H78C—C78B—H78D	108.6
C43—C44—H44	119.3		
			
C17—C12—C13—C14	0.7 (5)	C54—N8—C48—C53	−151.5 (2)
C12—C13—C14—C15	1.6 (5)	C42—N8—C48—C53	35.9 (3)
C13—C14—C15—C16	−1.6 (5)	C54—N8—C48—C49	29.4 (4)
C14—C15—C16—C17	−0.7 (5)	C42—N8—C48—C49	−143.2 (2)
C15—C16—C17—C12	3.0 (4)	C53—C48—C49—C50	−1.8 (4)
C15—C16—C17—C18	−175.8 (3)	N8—C48—C49—C50	177.3 (2)
C13—C12—C17—C16	−3.0 (4)	C48—C49—C50—C51	−0.1 (4)
C13—C12—C17—C18	175.7 (3)	C49—C50—C51—C52	1.4 (4)
Zn1—O3—C18—C19	−14.1 (4)	C49—C50—C51—C65	−175.0 (2)
Zn1—O3—C18—C17	164.23 (17)	C50—C51—C52—C53	−0.9 (4)
C16—C17—C18—O3	−26.9 (4)	C65—C51—C52—C53	175.5 (2)
C12—C17—C18—O3	154.4 (3)	C51—C52—C53—C48	−0.9 (4)
C16—C17—C18—C19	151.6 (3)	C49—C48—C53—C52	2.3 (4)
C12—C17—C18—C19	−27.1 (4)	N8—C48—C53—C52	−176.8 (2)
O3—C18—C19—C20	−4.6 (5)	C48—N8—C54—C55	−138.1 (3)
C17—C18—C19—C20	177.1 (3)	C42—N8—C54—C55	34.6 (4)
Zn1—O4—C20—C19	22.9 (4)	C48—N8—C54—C59	43.6 (4)
Zn1—O4—C20—C21	−157.62 (16)	C42—N8—C54—C59	−143.6 (3)
C18—C19—C20—O4	−0.5 (5)	C59—C54—C55—C56	0.3 (4)
C18—C19—C20—C21	−180.0 (2)	N8—C54—C55—C56	−177.9 (2)
O4—C20—C21—C22	−33.9 (3)	C54—C55—C56—C57	0.8 (4)
C19—C20—C21—C22	145.6 (3)	C55—C56—C57—C58	−1.4 (4)
O4—C20—C21—C26	141.5 (3)	C55—C56—C57—C70	177.0 (3)
C19—C20—C21—C26	−39.0 (4)	C56—C57—C58—C59	0.9 (4)
C26—C21—C22—C23	1.0 (4)	C70—C57—C58—C59	−177.5 (3)
C20—C21—C22—C23	176.6 (3)	C57—C58—C59—C54	0.2 (4)
C21—C22—C23—C24	−2.3 (5)	C55—C54—C59—C58	−0.8 (4)
C22—C23—C24—C25	1.9 (5)	N8—C54—C59—C58	177.4 (2)
C23—C24—C25—C26	−0.1 (6)	C44—C45—C60—C61	47.1 (4)
C24—C25—C26—C21	−1.3 (5)	C46—C45—C60—C61	−136.1 (3)
C22—C21—C26—C25	0.8 (4)	C44—C45—C60—C64	−130.2 (3)
C20—C21—C26—C25	−174.6 (3)	C46—C45—C60—C64	46.6 (4)
C32—C27—C28—C29	−1.3 (4)	C62—N9—C61—C60	0.2 (4)
C27—C28—C29—C30	0.3 (5)	Zn1—N9—C61—C60	175.8 (2)
C28—C29—C30—C31	1.3 (5)	C64—C60—C61—N9	0.9 (4)
C29—C30—C31—C32	−2.0 (5)	C45—C60—C61—N9	−176.6 (3)
C30—C31—C32—C27	1.0 (4)	C61—N9—C62—C63	−0.5 (4)
C30—C31—C32—C33	179.5 (3)	Zn1—N9—C62—C63	−175.9 (2)
C28—C27—C32—C31	0.6 (4)	N9—C62—C63—C64	−0.3 (5)
C28—C27—C32—C33	−177.7 (2)	C61—C60—C64—C63	−1.7 (4)
Zn2—O5—C33—C34	1.8 (4)	C45—C60—C64—C63	175.7 (3)
Zn2—O5—C33—C32	−178.96 (16)	C62—C63—C64—C60	1.5 (4)
C31—C32—C33—O5	−30.8 (4)	C52—C51—C65—C66	−32.2 (4)
C27—C32—C33—O5	147.5 (3)	C50—C51—C65—C66	144.0 (3)
C31—C32—C33—C34	148.5 (3)	C52—C51—C65—C69	149.8 (3)
C27—C32—C33—C34	−33.1 (4)	C50—C51—C65—C69	−34.0 (4)
O5—C33—C34—C35	−7.6 (5)	C67—N10—C66—C65	0.3 (4)
C32—C33—C34—C35	173.1 (2)	Zn2—N10—C66—C65	−176.1 (2)
Zn2—O6—C35—C34	8.4 (4)	C69—C65—C66—N10	−0.3 (4)
Zn2—O6—C35—C36	−170.81 (16)	C51—C65—C66—N10	−178.4 (2)
C33—C34—C35—O6	1.9 (4)	C66—N10—C67—C68	0.4 (4)
C33—C34—C35—C36	−178.9 (3)	Zn2—N10—C67—C68	177.0 (2)
O6—C35—C36—C41	−158.3 (3)	N10—C67—C68—C69	−1.2 (5)
C34—C35—C36—C41	22.5 (4)	C67—C68—C69—C65	1.1 (5)
O6—C35—C36—C37	21.6 (3)	C66—C65—C69—C68	−0.4 (4)
C34—C35—C36—C37	−157.6 (3)	C51—C65—C69—C68	177.7 (3)
C41—C36—C37—C38	−1.4 (4)	C58—C57—C70—C71	−27.2 (4)
C35—C36—C37—C38	178.7 (3)	C56—C57—C70—C71	154.5 (3)
C36—C37—C38—C39	0.4 (5)	C58—C57—C70—C74	152.7 (3)
C37—C38—C39—C40	1.0 (5)	C56—C57—C70—C74	−25.6 (4)
C38—C39—C40—C41	−1.4 (5)	C72—N11—C71—C70	0.7 (5)
C39—C40—C41—C36	0.4 (4)	C74—C70—C71—N11	−1.5 (5)
C37—C36—C41—C40	1.0 (4)	C57—C70—C71—N11	178.5 (3)
C35—C36—C41—C40	−179.1 (3)	C71—N11—C72—C73	0.7 (6)
C48—N8—C42—C47	−128.3 (3)	N11—C72—C73—C74	−1.1 (6)
C54—N8—C42—C47	58.8 (3)	C72—C73—C74—C70	0.2 (5)
C48—N8—C42—C43	52.4 (3)	C71—C70—C74—C73	1.0 (4)
C54—N8—C42—C43	−120.5 (3)	C57—C70—C74—C73	−179.0 (3)
C47—C42—C43—C44	−2.2 (4)	C78A—O7A—C75A—C76A	31.5 (15)
N8—C42—C43—C44	177.1 (2)	O7A—C75A—C76A—C77A	−22.7 (14)
C42—C43—C44—C45	1.7 (4)	C75A—C76A—C77A—C78A	6.4 (17)
C43—C44—C45—C46	−0.4 (4)	C75A—O7A—C78A—C77A	−28.1 (18)
C43—C44—C45—C60	176.5 (2)	C76A—C77A—C78A—O7A	12.8 (19)
C44—C45—C46—C47	−0.4 (4)	C78B—O7B—C75B—C76B	−33 (3)
C60—C45—C46—C47	−177.3 (3)	O7B—C75B—C76B—C77B	19 (3)
C43—C42—C47—C46	1.4 (4)	C75B—C76B—C77B—C78B	1 (3)
N8—C42—C47—C46	−178.0 (2)	C75B—O7B—C78B—C77B	35 (3)
C45—C46—C47—C42	−0.1 (4)	C76B—C77B—C78B—O7B	−21 (4)

Symmetry codes: (i) −*x*+1, −*y*+1, −*z*+2; (ii) −*x*, −*y*+1, −*z*+1.

### Hydrogen-bond geometry (Å, º)

*Cg*1, *Cg*2, *Cg*3 and *Cg*4 are the centroids of the N10/C65–C69, C54–C59, C36–C41 and N11/C70–C74 rings, respectively.

**Table:**

*D*—H···*A*	*D*—H	H···*A*	*D*···*A*	*D*—H···*A*
C13—H13···O7*A*	0.95	2.47	3.197 (7)	134
C40—H40···*Cg*1^iii^	0.95	2.74	3.594 (3)	150
C43—H43···*Cg*2^iv^	0.95	2.78	3.572 (3)	142
C68—H68···*Cg*3^v^	0.95	2.65	3.513 (3)	152
C75*B*—H75*C*···*Cg*4^iv^	0.99	2.78	3.649 (17)	146

Symmetry codes: (iii) *x*, *y*, *z*−1; (iv) *x*, −*y*+1/2, *z*−3/2; (v) *x*, −*y*+1/2, *z*−1/2.
